# Effects of Heart Rate vs. Speed-Based High Intensity Interval Training on Aerobic and Anaerobic Capacity of Female Soccer Players

**DOI:** 10.3390/sports5030057

**Published:** 2017-08-04

**Authors:** Hamid Arazi, Abbas Keihaniyan, Amin EatemadyBoroujeni, Amir Oftade, Sheida Takhsha, Abbas Asadi, Rodrigo Ramirez-Campillo

**Affiliations:** 1 Department of Exercise Physiology, Faculty of Sport Sciences, University of Guilan, Rasht 1438, Iran; abbas.keihaniyan@yahoo.com (A.K.); Abbas_asadi1175@yahoo.com (A.A.); 2 Department of Exercise Physiology, Faculty of Sport Sciences, University of Isfahan; Isfahan 81746, Iran; eatemady@gmail.com; 3 Department of Exercise Physiology, Faculty of Sport Sciences, University of Shahid Beheshti, Tehran 19834, Iran; Amiroftade@yahoo.com; 4 Department of Exercise Physiology, Faculty of Sport Sciences, Isfahan Islamic Azad University, Khorasgan Branch, Khorasgan 81595-158, Iran; sheida.takhsha@yahoo.com; 5 Roudbar Branch, Islamic Azad University, Roudbar, 446161-4579, Iran; 6 Department of Physical Activity Sciences, Research Nucleus in Health, Physical Activity and Sport, Universidad de Los Lagos, Osorno 5290000, Chile; r.ramirez@ulagos.cl

**Keywords:** VIFT, women, football, power

## Abstract

The purpose of this study was to compare the effects of two types of high-intensity interval training (HIIT) programs on aerobic and anaerobic capacity of female soccer players. Regional-level female athletes were randomly divided into heart rate-based HIIT (*n* = 8; age 23.4 ± 1.1 year) and speed-based HIIT groups (*n* = 8; age 23.4 ± 1.3 year). Athletes trained three days per week for six weeks. Before and after training, each athlete’s performance was assessed directly through the Hoff test, 30-15 Intermittent Fitness Test (VIFT), and repeated-sprint ability test (RAST); maximal oxygen consumption (VO_2_max), power and fatigue were estimated indirectly. Both experimental groups improved power, fatigue index and VO_2_max after training (*p* < 0.05). It was noteworthy that the speed-based group had greater gains in minimal power (effect size (ES): 3.99 vs. 0.75), average power (ES: 2.23 vs. 0.33), and fatigue index (ES: 2.53 vs. 0.17) compared to heart rate-based group (*p* < 0.05). In conclusion, both heart rate-based and speed-based HIIT induced meaningful improvements in power, VO_2_max, and fatigue index in female soccer players, although the speed-based HIIT group achieved greater gains in power and fatigue index compared to the heart rate-based group.

## 1. Introduction

Soccer is an intermittent sport that requires different physiological components [1,2]. The dominant energy system in soccer is the aerobic system, with soccer players covering up to 12 kilometers per game [3,4,5] at ~75% of VO_2_max [2,6]. It has been reported that improvements in aerobic capacity (i.e., VO_2_max, running economy and lactate threshold) are in line with increases in players’ physical fitness, technique and tactical performance [1,7]. Moreover, the capacity to produce varied powerful actions during a 90-min game is associated with high aerobic capacity [1,2]. In addition to aerobic capacity, several short-duration and maximal-intensity movements in soccer depends on anaerobic capacity, were athletes perform 1000 to 1400 of such movements during a game [3,4]. Therefore, anaerobic and aerobic capacities maybe critical to soccer performance.

An effective training method to improve these variables is high-intensity interval training (HIIT), with positive effects on both aerobic and anaerobic fitness [8]. The literature, particularly with reference to high-intensity interval training (HIIT), has recently been reviewed [9]. Since one of the chief barriers to broad public participation in exercise programs is a perceived lack of time [10], one of the appeals of HIIT training has been that it potentially represents a more time-efficient way of accomplishing the adaptive goals of exercise training. HIIT may improve soccer players’ VO_2_max [1,8,11], power [11,12], fatigue resistance [13], stroke volume [14], oxidative and glycolytic enzyme activities [11], lactate tolerance [15,16], motor efficiency and lactate utilization [1], with reduced exercise time requirements. However, optimum HIIT design is elusive, especially regarding intensity prescription [2] and practical strategies to control it.

Prescription of HIIT based on heart rate (HR) or running speed are common ways to control the intensity for soccer players.

Speed-based training, or maximal running velocity (VIFT) training, is a graded intermittent and shuttle field training. The use of VIFT has been shown to be more accurate than individual VO_2_max (i.e., the lowest running velocity that elicits maximal oxygen uptake during a continuous graded test) for getting players with different physiological profiles to a similar level of cardiorespiratory demand and, thus, for standardizing training content at a team level [8]. In contrast, HR-based training (i.e., Hoff method) proposed a specific circuit with jumps, running backwards and changing directions whilst dribbling the ball, which has been shown to be related to match performance [8]. This circuit is also useful to determine maximal oxygen consumption [11] and maximal heart rate (HRmax) [11], but the maximum distance covered in the Hoff circuit is not correlated with aerobic endurance determined on the treadmill [16]. Use of HR (i.e., Hoff method), although probably the most commonly used method to prescribe the intensity for HIIT, is limited by the athlete’s psychological status and ability to regulate running intensity, and HR is usually poorly associated with metabolic demands [17]. Alternatively, speed-based HIIT prescriptions (i.e., 30-15 Intermittent Fitness Test) are also common, and have been shown to be relevant for players with different fitness levels [18].

Moreover, investigating the effects of these methods on enhancing physiological variables in female soccer players is paramount. Exercise training in female players may improve their maximal-intensity exercise and endurance. However, further investigation in this population is required, especially with regard to factors that might be mediating the effects of HIIT on aerobic and anaerobic capacity adaptations. To our knowledge, there are no previous studies that have examined the influence of HR vs. speed-based HIIT training on physiological adaptations in female soccer players. To help clarify practical strategies for optimal intensity prescription, the purpose of this study was to compare the effects of HR-based and speed-based intensity sessions for HIIT on aerobic and anaerobic capacity of female soccer players.

## 2. Materials and Methods

### 2.1. Study Design

Female soccer players participated in the study and were randomly divided into two HIIT groups with different intensity prescription methods: HR-based and speed-based. Before and after six weeks of training, athletes performed the Hoff, 30-15 Intermittent Fitness Test (VIFT), and RAST tests to determine the effects of HIIT training on aerobic and anaerobic capacities of female soccer players. Both HR-based and speed-based training sessions were performed at the same time of day (i.e., morning).

### 2.2. Participants

Sixteen healthy female soccer players from a semi-professional soccer academy with similar training habits and loads volunteered to participate in this study, and were randomly divided into two training groups (Table 1). In order to ensure no subjects had any orthopedic or health related conditions that could preclude them from participating in training and measurements, all subjects underwent a supervised screening undertaken by a physician. Exclusion criteria included subjects with potential medical problems; subjects with a history of ankle, knee, or back pathology in the three months before the study; subjects with medical or orthopedic problems that compromised their participation or performance; subjects who had had any lower extremity reconstructive surgery in the past two years; and subjects with unresolved musculoskeletal disorders or a pregnancy period. The study was conducted in accordance with the Institutional Ethics Review Committee from the University.

### 2.3. Testing Procedures

All tests were performed one week before and after 6 weeks of HIIT in the morning (i.e., 9 to 11 a.m.). Before testing, subjects performed a 10-min general warm-up protocol consisting of sub-maximal running (i.e., 5-min), active stretching (i.e., 5-min), and three submaximal vertical jumps. Prior to each test, the players performed 2–3 submaximal trials for familiarization.

#### 2.3.1. Hoff Test

**Figure 1:**
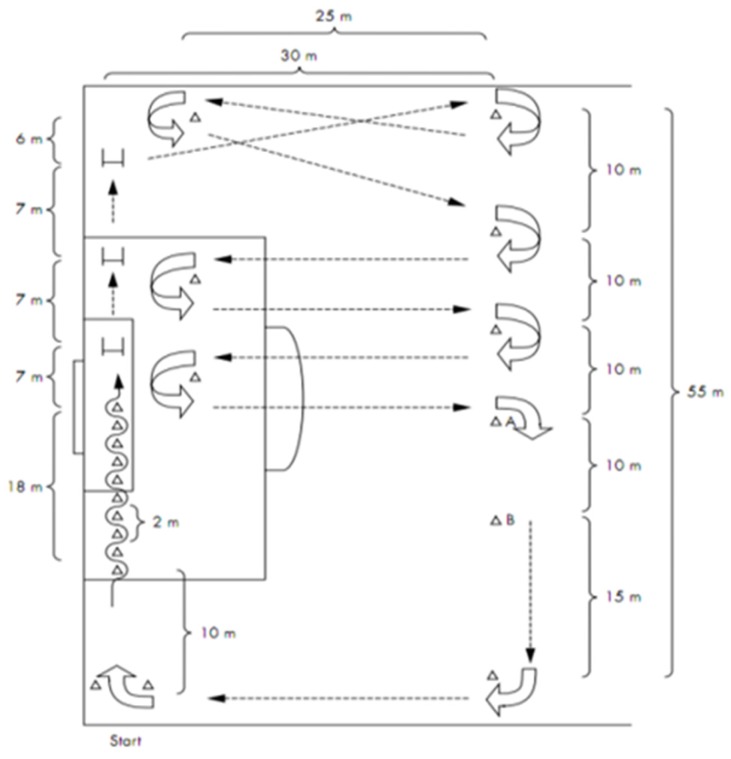
Hoff test (details in text).

The procedure of this test is described in detail elsewhere [19]. Briefly, according to Figure 1, from the start point, players dribbled through the first 10 cones, then jumped three 30-cm height cones, then dribbled through 8 cones as fast as possible toward point A. Athletes then run backwards from point A to point B, and after point B players run forward again to the starting point. Each athlete has 10 min to complete as many laps as possible. Total distance (m) and estimated VO_2_max were measured as markers of specific endurance of soccer players, regarding previously described [19,20]. Players are to dribble the ball during the test. During the test, subjects were encouraged to perform at maximal effort.

#### 2.3.2. V_IFT_ Test

Buchheit [21] described the VIFT previously. Briefly, this test consists of 30-s shuttle runs interspersed with 15-s passive recovery periods. Velocity was set at 8 km/h for the first 30-s run and was increased by 0.5 km/h every 45-s stage thereafter. Calculation of targeted distances to run during each 30-s period took into account the fact that the effort to turn is increased when running speed is increased. An empirical value of 0.7 s was subtracted from the 30-s running periods for each change of direction. For example, at 11.5 km/h, one would cover 96 m in a 30-s straight line run, but covering the same distance over a 40-m shuttle distance requires two changes of direction taking up to 0.7 s, which brings the corrected distance run to 91.6-m. The subjects had to run back and forth between two lines set 40-m apart at a pace governed by a pre-recorded beep at appropriate intervals that helped them adjust their running speed by entering into 3-m zones at each end of the running area and in the middle of the area, while the short beep sounds. During the 15-s recovery period, the subjects walked in the forward direction to join the closest line (at the middle or at one end of the running area, depending on where the previous run stopped) from where they started the next run stage. Performance in this test was expressed as estimated VO_2_max (mL∙kg^−1^∙min^−1^), speed (km∙h^−1^) and time (min). During the test, subjects were orally encouraged to perform at maximal effort [18].

$$\begin{array}{cc} {\mathrm{VO}}_{2\max}= & 28.3-2.15\times \mathrm{Sex}\left(\mathrm{Male}=1,\mathrm{Female}=2\right)-0.741\left(\mathrm{Age}\right)-0.357\left(\mathrm{Weight}\right)\\ & +0.0586\left(\mathrm{Age}\right)\left(\mathrm{VIFT}\;\left[\mathrm{speed},\;\mathrm{km}/h\right]\right)+1.03\left(\mathrm{VIFT}\;\left[\mathrm{speed},\;\mathrm{km}/h\right]\right) \end{array}$$

#### 2.3.3. RAST Test

This test was used to measure athletes’ anaerobic performance ability, including minimum, maximum and average power, and fatigue index. Athletes run 35-m intervals, six times, with 10 s of rest between each interval. Power and fatigue index were calculated as previously suggested [21]. To estimate power (W) and fatigue index (W/s), a previously established equations (power = $\frac{\mathrm{weight}\times \mathrm{distance}\;2}{\mathrm{time}\;3}$ , fatigue index = $\frac{\mathrm{maximal}\;\mathrm{power}-\mathrm{minimal}\;\mathrm{power}}{\mathrm{total}\;\mathrm{time}\;\mathrm{of}\;\mathrm{six}\;\mathrm{repetitions}}$) were used.

### 2.4. Training Program

Athletes performed regular soccer practices three days per week for 60–70 min on Sunday, Thursday and Friday, and participated in their HIIT programs three days per week (Saturday, Monday, and Wednesday) which added to their regular training load. All subjects in the present study were required to complete all training sessions. A trained researcher monitored training sessions in order to ensure that all exercises were performed correctly with the appropriate rest intervals.

#### 2.4.1. HR-Based HIIT Protocol

Athletes run at 90% of maximal HR for 90 s and then walk for 90 s, with the pattern repeated during 7.5 min. Then athletes rested passively for 2 min. This sequence was repeated three times, for twelve 90-s runs per session. HR was recorded during training sessions (Heart rate monitor, Acentas pulse meter, BM-CS5EU, Beijing, China). Regarding the principle of overload, although not controlled, over time it was perceived that athletes ran longer distances while maintaining 90% of maximal HR for 90 s.

#### 2.4.2. Speed-Based HIIT Protocol

After determining maximal speed during the VIFT test, 90% of the maximal speed and traveled distance for each athlete was selected. Players had to get to the cone with every beep (beep speed was designed based on 10 km/h). Athletes run at this speed for 90 s, and then walk for 90 s. This activity-recovery pattern was repeated for 7.5 min, with passive rest for 2 min. This sequence was repeated two times. Regarding the principle of overload, the velocity increased 5% every two weeks [20].

### 2.5. Statistical Analysis

IBM SPSS version 22 software was used to analyze the data. Normality of the collected data was evaluated by the Shapiro–Wilk test after the verification of data normality, 2 × 2 ANOVA was used to determine the effects of training on dependent variables. The magnitude of the effect size statistics was considered trivial <0.2; small, 0.2–0.49; moderate, 0.5–0.79; large, 0.8–1.3; very large >1.30 [22]. The effect size is reported in conjunction with the 95% confidence limits (CI) for all analyzed measures. The level of significance was set at *p* ≤ 0.05. The statistical tests were performed using the SPSS statistical package version 16 (Chicago, IL, USA).

## 3. Results

There were no significant differences between the groups at pre-training. No significant groupings by time interaction were observed after HIIT for the maximal power (F = 0.009, P = 0.653), Hoff test traveled distance (F = 0.08, P = 0.43), VO_2max_ in Hoff (F = 0.11, P = 0.88), VIFT (F = 0.93, P = 0.13), VIFT speed (F = 0.319, P = 0.586) and time (F= 1.1, P = 0.7) tests. After 6-week HIIT, the speed-based group indicated greater gains in minimal power (F = 5.08, P = 0.04), average power (F = 32.12, P = 0.003), and fatigue index (F = 28.32, P = 0.002) compared with the HR-based group (Table 2).

## 4. Discussion

Strength and conditioning coaches, to improve fitness level in soccer players, have extensively used HIIT. HIIT may induce both central and peripheral physiological adaptations [8]. However, to the authors’ knowledge the comparison of HR-based and speed-based intensity prescription during HIIT have never been examined in female soccer players. Therefore, the purpose of this study was to compare the effects of HR-based and speed-based intensity prescriptions for HIIT on aerobic and anaerobic capacity of female soccer players. Main results indicate that both intensity prescription methods are practical and effective to improve aerobic and anaerobic fitness variables in female soccer players (except maximal power for both groups, and fatigue index and average power for the HR-based HIIT group). However, speed-based intensity prescription induced greater improvements in power (i.e., average and minimal) and fatigue resistance compared to the HR-based prescription. In line with current findings, a number of studies have shown that HIIT improve VO_2_max and anaerobic capacities [16,17,18,23,24,25]. For example, Sperlich et al. [26] and Helgerud et al. [1] reported 7 to 11% improvements in VO_2_max after 5 to 8 weeks of HIIT in soccer players. HIIT may induce large adaptive responses by virtue of recruiting a broader population of muscle fibers and enhance cardiorespiratory signaling, resulting in VO_2_max and anaerobic capacity gains [15,25,27]. There are several mechanisms to enhance VO_2_max after HIIT including increases in muscle oxidation and buffering capacities, enhancements of PGC-1α, which plays an important role in mitochondrial gene transcription and other biochemical changes [26,27,28,29].

Regarding the important role of power and fatigue index during soccer games, both HR-based and speed-based HIIT prescription methods induced meaningful improvements in power-related performance and fatigue index in soccer; however, no previous studies reported these variables in female soccer players following HIIT. During cycling sprint interval training (SIT), Burgomaster et al. [30] reported that 6 weeks SIT induced 17% improvements in power output. In addition, Zieman et al. [31] examined the effects of 6 × 90 s SIT with 80% VO_2_max on power of physically active men and found significant increases. The possible mechanisms for the enhancement of anaerobic variables after HIIT could be the involvement of ATP-PC and anaerobic glycolis systems during periods of trials that involve repeated high-intensity sprints with relatively short recovery intervals, resulting in anaerobic enzyme adaptations [26]. Based on the findings of the present study, it appears that speed-based HIIT induced greater efficiency compared to HR-based (Hoff approach) methods in aerobic and anaerobic adaptation tests. The greater adaptive responses by VIFT-based HIIT vs. Hoff may be related to better regulation of running intensity in the latter, including the use of perceived exertion during training. Alternatively, the inability of HR to determine their association with metabolic demands and to inform running intensities above vVO_2_max [17,18] may also help to explain the results from this study. However, more studies are necessary to determine the influence of each variable and also determine role of each variable to enhance training adaptation following HIIT.

## 5. Conclusions

This is the first study that examined the effects of HR-based and speed-based intensity prescription for HIIT on aerobic and anaerobic capacity of female soccer players. The results of this study showed that both intensity prescription methods are practical and effective for improving aerobic and anaerobic fitness. However, speed-based intensity prescription induced greater improvements in power and fatigue resistance compared to HR-based prescription.

## Figures and Tables

**Table 1:** Subject characteristics.

Characteristics	HR-Based (*n* = 8)	Speed-Based (*n* = 8)
Age (years)	22.4 ± 1.1	22.4 ± 1.3
Weight (kg)	59.1 ± 1.3	55.9 ± 2.3
Height (cm)	170.9 ± 2.6	165.1 ± 3.2
BMI (kg∙m^−2^)	20.3 ± 0.8	20.6 ± 0.9
VO_2_max (mL∙kg^−1^∙min^−1^)	50.3 ± 1.8	50.6 ± 2.2
Soccer experience (years)	10.1 ± 2.8	10.3 ± 3.1
Weekly soccer training (time, hour)	4.5 ± 0.2	4.5 ± 0.3

Values are mean ± SD.

**Table 2:** Changes in aerobic and anaerobic capacity for the HR-based (*n* = 8) and speed-based HIIT groups (*n* = 8). Values are reported as mean ± SD.

Variables	Before	After	Effect Size	Confidence Limits (CI)
**Maximal Power (Watts)**				
HR-based	562 ± 32.9	572 ± 35.6	0.29	1.25 to −0.71
Speed-based	568 ± 21.4	579 ± 23.3	0.1	1.07 to −0.89
**Minimal Power (Watts)**				
HR-based	289 ± 17.3	302 ± 15.6 *	0.75	1.72 to −0.3
Speed-based	309 ± 13.0	366 ± 15.3 *^,†^	3.99	5.41 to 2.14
**Average Power (Watts)**				
HR-based	425 ± 21.5	432 ± 20.3	0.33	1.29 to −0.68
Speed-based	430 ± 13.8	464 ± 16.8 *^,†^	2.23	3.33 to 0.88
**Fatigue index (W/s)**				
HR-based	8.3 ± 0.8	8.1 ± 0.8	0.17	1.14 to −0.82
Speed-based	8.0 ± 0.5	6.7 ± 0.5 *^,†^	2.53	3.67 to 1.11
**Hoff test traveled distance (m)**				
HR-based	1308 ± 120	1630 ± 234 *	1.37	2.77 to −0.51
Speed-based	1311 ± 139	1468 ± 165 *	1.02	2.50 −0.07
**VO_2_max in Hoff test (mL∙kg^−1^∙min^−1^)**				
HR-based	48.6 ± 6.2	54.8 ± 10.6 *	0.71	1.67 to −0.34
V_IFT_	49.2 ± 4.1	53.6 ± 11.6 *	0.51	1.47 to −0.52
**VO_2_max in V_IFT_ test (mL∙kg^−1^∙min^−1^)**				
HR-based	50.6 ± 7.7	53.6 ± 8.2 *	0.38	1.34 to −0.63
Speed-based	50.3 ± 6.1	59.0 ± 6.5 *	1.39	2.39 to −0.23
**V_IFT_ speed (km∙h^−1^)**				
HR-based	12.0 ± 3.5	13.6 ± 3.0 *	0.49	1.46 to −0.53
Speed-based	12.7 ± 3.5	16.3 ± 3.7 *	1.01	1.99 to −0.08
**V_IFT_ time (min)**				
HR-based	7.1 ± 0.5	9.0 ± 0.5 *	3.89	5.20 to 2.07
Speed-based	7.6 ± 0.5	13.5 ± 0.7 *	4.3	6.45 to 1.27

* denotes significant differences between baseline and post training values (*p* ≤ 0.05); ^†^ denotes significant differences between the experimental groups at post training (*p* ≤ 0.05).
